# Round Ligament Leiomyoma Presenting as an Incarcerated Inguinal Hernia: Case Report and Review of the Literature

**DOI:** 10.1155/2016/9380212

**Published:** 2016-04-06

**Authors:** Marc Najjar, Marc Mandel

**Affiliations:** Department of Surgery, College of Physicians and Surgeons, Columbia University, New York, NY 10032, USA

## Abstract

Leiomyomas are common benign gynecologic tumors occurring in up to 30% of women. Round ligament leiomyomas however are very rare and, if symptomatic, can present as an inguinal hernia. We report the case of a 47-year-old woman who presented with an irreducible inguinal mass consistent with an incarcerated hernia. Intraoperatively, the mass was found to be a round ligament leiomyoma, a diagnosis that was confirmed by histopathology following excision of the mass. Although rare, round ligament leiomyomas should be part of the differential diagnosis of an inguinal hernia in females.

## 1. Introduction

The round ligament (RL), the embryological equivalent of the gubernaculum testis in females, is composed mainly of smooth muscle fibers and extends from the uterus to the labia majora passing through the inguinal canal. RL tumors are rare but whenever they occur, leiomyomas are the most common lesions [1, 2]. Leiomyomas are the most common gynecologic tumors with a prevalence of 20–30% among women and are most commonly found in the uterus [3–5]. RL leiomyomas however are very rare, occur mainly in premenopausal women, and are most often asymptomatic. We report a rare case of extraperitoneal round ligament leiomyoma that presented as an incarcerated inguinal hernia.

## 2. Case Report

**Figure 1:**
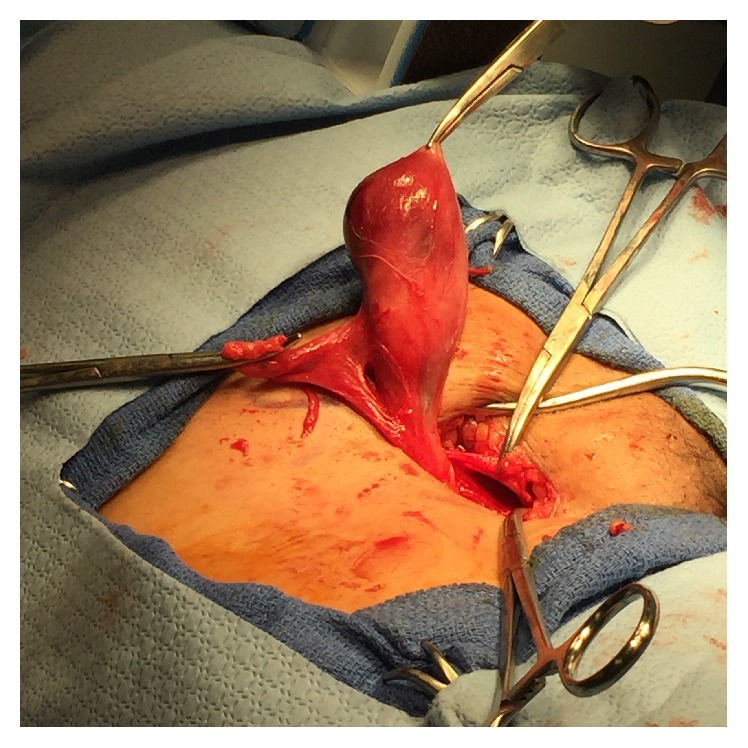
Mass arising from the right round ligament.

**Figure 2:**
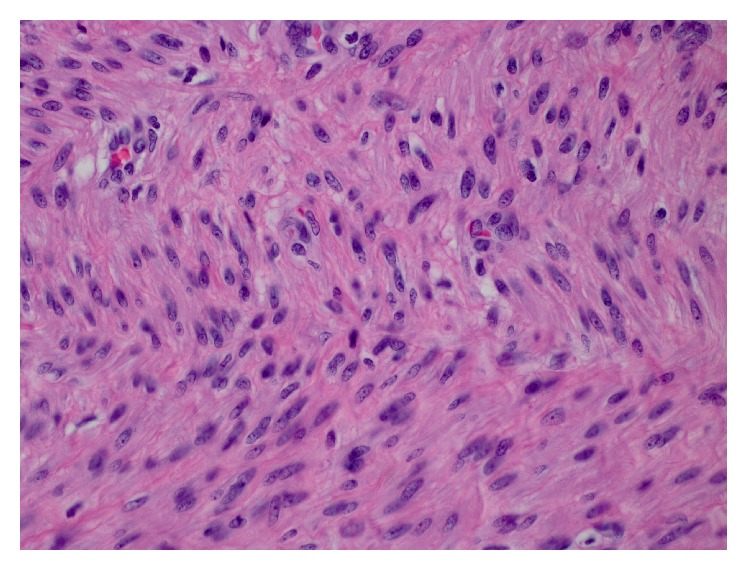
Medium power magnification H&E stain of round ligament leiomyoma showing spindle cells with “cigar shaped” nuclei and “herringbone” pattern often seen in smooth muscle tumors. No evidence of perinuclear vacuolization, hypercellularity, or cytological atypia.

**Figure 3:**
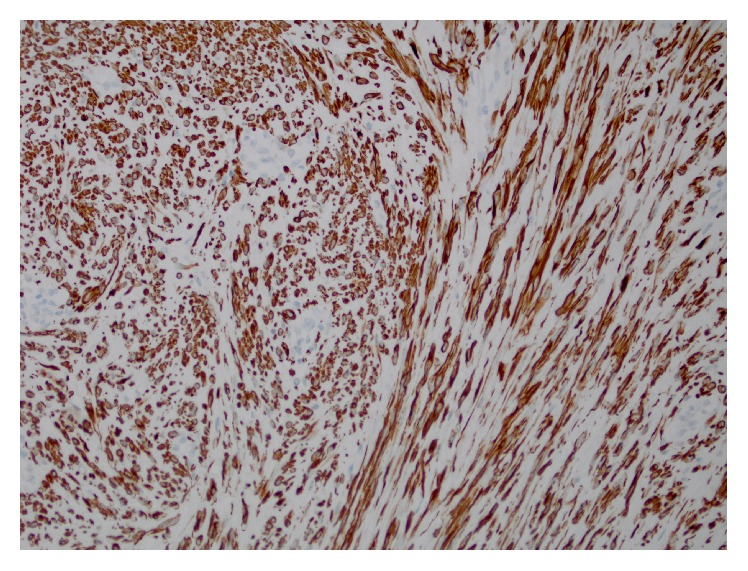
Immunohistochemical stain for smooth muscle desmin.

A 47-year-old G1P1 African American woman presented with a right groin mass. The mass was noticed around four months ago and has been intermittently painful. The patient stated that the mass has grown in size recently. She denied any associated gastrointestinal or urinary symptoms and denied any recent weight loss, fever, or chills. She had no past medical history, no history of uterine leiomyomas, and no long-term estrogen use. Her past surgical history was significant for a Cesarean section followed by an abdominoplasty and a laparoscopic appendectomy. On physical exam, the patient's groins were asymmetric in the standing position; a bulge was visible in the right inguinal area. Upon palpation, the mass was round and firm measuring around 4 cm. Tenderness was only elicited with deep palpation. The mass was only partially reducible with pressure and with the patient in the supine position. A working diagnosis of incarcerated right inguinal hernia was made and the patient was scheduled for an elective surgical repair. Blood workup was within normal limits and no imaging was obtained. Under general anesthesia, a transverse groin incision was made and the external oblique fascia opened. A 4 × 3 × 2 cm firm well-circumscribed egg-shaped mass adherent to the RL was encountered; no hernia sac was seen (Figure 1). The mass was removed completely and sent for frozen section pathology, which revealed a spindle cell tumor with “cigar shaped” nuclei and “herringbone” pattern often seen in smooth muscle tumors. No evidence of perinuclear vacuolization, hypercellularity, or cytological atypia was found (Figure 2). The findings were consistent with the diagnosis of leiomyoma. Immunohistochemical stain for desmin later confirmed the diagnosis (Figure 3).

Following complete excision of the round ligament and lesion, it was noted that the internal ring was vacant, as it no longer had anything to obturate it. Additionally, the floor of the inguinal canal was weakened from the dissection. Therefore, a formal hernia repair was performed which included closure of the ring and placement of a lightweight mesh patch.

## 3. Discussion

The RL, the embryological equivalent of the gubernaculum testis in females, is composed mainly of smooth muscle fibers and extends from the uterus to the labia majora passing through the inguinal canal. Leiomyomas are benign smooth muscle tumors found in 20–30% of women older than 35 [3–5]. They are estrogen sensitive owing to the presence of estrogen receptors on smooth muscle cells [6]. Risk factors associated with their growth include age, early menarche, late menopause, nulliparity, estrogen replacement therapy, and obesity. RL tumors are very rare; however, when present, leiomyomas are the most common tumors followed by endometriomas and mesothelial cysts [7–9]. Half of RL leiomyomas are extraperitoneal and are usually asymptomatic; only a few cases presenting as an inguinal mass mimicking an inguinal hernia have been reported in the literature [1, 2, 5, 10–15]. Lesions are usually unilateral, are most commonly found on the right side, and range on average in size from 3 to 4 cms [7]. The case we described presented similarly to the ones previously reported. Transformation of leiomyomas to leiomyosarcomas is extremely rare and only one case of RL leiomyosarcoma has been reported in the modern era [10].

Inguinal masses in women have a long differential diagnosis list including inguinal and femoral hernias, lymphadenopathy, psoas abscess, femoral artery aneurysm, hydrocele of canal of Nuck, leiomyoma, leiomyosarcoma, endometrioma, and other round ligament lesions. Preoperative imaging can be helpful in diagnosing rare causes of inguinal masses but are very rarely performed. However, when obtained, a CT scan can be useful in diagnosing a RL leiomyoma which appears as a well-circumscribed lesion with bright and heterogeneous enhancement, an appearance typical of but not specific to leiomyomas [5, 16]. Definitive diagnosis and treatment of extraperitoneal RL leiomyomas are obtained through surgical excision. No recurrences of the lesions have been reported to date.

In cases of RL leiomyomas, a fine needle or a core needle biopsy could potentially confirm the diagnosis preoperatively [17]; however, based on the physical exam findings, a working diagnosis of incarcerated right inguinal hernia is often made. Moreover, considering how commonly incarcerated inguinal hernias are encountered compared to the rarity of RL leiomyomas presenting as inguinal hernias it would be impractical to biopsy each and every patient especially in the setting of low suspicion such as the presented case.

As described above, we felt it necessary to perform a formal hernia repair following the excision as the internal ring was vacated and the floor was weakened. It is our recommendation that whenever a lesion is excised from the inguinal canal and the round ligament removed, a formal inguinal hernia repair should be performed, preferably with mesh, in order to complete the procedure and prevent a hernia from occurring. However, other types of hernia repair without mesh could also be considered depending on surgeons' preference.
